# Personalized Response of Parkinson’s Disease Gut Microbiota to Nootropic Medicinal Herbs In Vitro: A Proof of Concept

**DOI:** 10.3390/microorganisms11081979

**Published:** 2023-08-01

**Authors:** Christine Tara Peterson, Stanislav N. Iablokov, Dmitry A. Rodionov, Scott N. Peterson

**Affiliations:** 1 Center of Excellence for Research and Training in Integrative Health, Department of Family Medicine, School of Medicine, University of California San Diego, La Jolla, CA 92023, USA; 2 Phenobiome Inc., Palm Springs, CA 92262, USA; stan@phenobiome.com; 3 Bioinformatics and Structural Biology Program, Sanford Burnham Prebys Medical Discovery Institute, La Jolla, CA 92037, USA; rodionov@sbpdiscovery.org; 4 Tumor Microenvironment and Cancer Immunology Program, Sanford Burnham Prebys Medical Discovery Institute, La Jolla, CA 92037, USA; speterson@sbpdiscovery.org

**Keywords:** gut microbiota, Ayurveda, nootropic herbs, personalized medicine, Parkinson’s disease, genome-wide metabolic reconstruction, metagenomics

## Abstract

Parkinson’s disease (PD) is a neurodegenerative disease characterized by the loss of dopaminergic neurons. Although the etiology of PD remains elusive, it has been hypothesized that initial dysregulation may occur in the gastrointestinal tract and may be accompanied by gut barrier defects. A strong clinical interest in developing therapeutics exists, including for the treatment of gut microbiota and physiology. We previously reported the impact of healthy fecal microbiota anaerobic cultures supplemented with nootropic herbs. Here, we evaluated the effect of nootropic Ayurvedic herbs on fecal microbiota derived from subjects with PD in vitro using 16S rRNA sequencing. The microbiota underwent substantial change in response to each treatment, comparable in magnitude to that observed from healthy subjects. However, the fecal samples derived from each participant displayed unique changes, consistent with a personalized response. We used genome-wide metabolic reconstruction to predict the community’s metabolic potential to produce products relevant to PD pathology, including SCFAs, vitamins and amino acid degradation products. These results suggest the potential value of conducting in vitro cultivation and analyses of PD stool samples as a means of prescreening patients to select the medicinal herbs for which that individual is most likely to respond and derive benefit.

## 1. Introduction

Recent investigations suggest that altered gut microbiota and microbial metabolites, such as butyrate, LPS and amyloid, are associated with neurodegenerative diseases such as Parkinson’s Disease (PD). Establishing causality and the precise mechanisms through which gut microbiota contribute harmful and beneficial effects to PD pathology is an area of great interest, requiring additional study. Burgeoning investigation of dysbiotic signatures in PD has revealed a variety of features of the PD gut microbiome that differ compared to healthy control subjects, including the reduced relative abundance of several butyrate producers [1,2,3,4,5]. Microbiota generate butyrate as a result of sugar and amino acid fermentation, resulting in the expansion of anti-inflammatory Tregs and beneficial neuroactive effects [6,7,8]. Microbiota-dependent toxic effects may be linked to the increased intestinal permeability in these patients because butyrate is the primary energy source for enterocytes and actively regulates gut barrier integrity [2,9,10,11]. Several additional studies reporting relationships between gut microbiota and disease phenotypes in PD subjects have been conducted [1,12,13,14,15,16].

Gut and systemic inflammation and the activation of microglia and/or enteric glia are also hypothesized to contribute to α-syn misfolding and aggregation [17]. Alpha synucleinopathy has been observed in the enteric nervous system prior to disease onset and subsequent CNS involvement [18,19]. Increased intestinal permeability and disruption of the blood–brain barrier is observed in PD, which may facilitate pathological communication between the gut microbiota and the immune cells of the CNS [20,21,22]. As in other disease conditions, gut microbiota may provide beneficial effects and, when dysbiotic, may promote disease phenotypes. In a mouse model of PD, mice receiving the antibiotic minocycline and LPS displayed increased dopaminergic neuron survival rates compared to mice treated with LPS alone. In mice overexpressing α-syn, it was established that motor symptoms, Lewy body pathology, microglial cell activation and neuroinflammation were all negatively impacted by the presence and activities of the gut microbiota [19]. Germ-free mice colonized with PD patient stool exhibit greater motor dysfunction compared to mice colonized with healthy donor stool [19]. These studies highlight the pathogenic potential of the PD-driven dysbiosis of gut microbiota, providing evidence to support a model of PD involving reduced butyrate and other factors that promote elevated gut permeability and subsequent LPS translocation across the gut epithelium and access to circulation, thereby worsening motor defects [23]. By contrast, in PD animal models, β-hydroxybutyric acid treatment reduced LPS-mediated microglial activation and protected dopaminergic neurons [24,25]. Local inflammatory responses to LPS reinforce gut barrier defects in a positive feedback loop [26]. Increased systemic LPS concentration leads to chronic microglial activation and accelerates α-syn misfolding and post-translational modifications, resulting in decreases in dopaminergic neurons and motor function.

Drug discovery for PD is an active field of investigation that has not advanced sufficiently beyond controlling dopamine levels in patients. Historically, natural products have been featured prominently in drug discovery [27]. Renewed interest in natural products derived from the study of gut microbiota have emerged due to the impressive array of bioactive secondary metabolites that may positively and negatively alter host physiology [28]. Nervine herbs, such as Bacopa, are cornerstone treatments for PD, memory enhancement and improved cognition in integrative and traditional medicine such as Ayurveda [29]. Bacosides A and B are in high abundance, and triterpenoid saponins implicated in some neuropharmacological effects of Bacopa cross the blood-brain barrier [30,31]. In MPTP-induced mouse models of PD, Bacopa treatment generated neuroprotective effects on nigrostriatal dopaminergic neurons and modulated oxidative stress and apoptosis [32,33].

We previously reported the impact of these 10 nootropic medicinal herbs from Ayurvedic medicine on fecal microbiota in vitro. We noted the substantial modulatory capacity of these herbs involving the altered relative abundance of over one third of profiled species in healthy subjects [34]. We quantified the sugar composition of these medicinal herbs and concluded that that the sugar content served as the driving force for each herb’s modulatory effects. In separate studies, we established that the impact of medicinal herbs in humans is personalized [35,36]. It is possible that the inter-personal differential response of microbiota to medicinal herbs is due to the strain variation of bacterial species that are herb-responsive in some individuals but not others. In this study, we conducted a small-scale proof of concept to assess the extent to which the microbiota response to 10 nootropic medicinal herbs from Ayurveda is personalized. The results of this analysis suggest that the in vitro cultivation and sequencing of fecal samples may provide a means to evaluate the treatments most likely to have a beneficial effect in treating host phenotypes and symptoms. Here, we demonstrate this concept in the context of PD.

## 2. Materials and Methods

### 2.1. Study Participants and Sample Collection

Three PD stool samples were obtained through the Center for Integrative Health, University of California, San Diego School of Medicine. Twelve healthy, English-speaking women and men aged 30–60 years that had previously adhered to a vegetarian or vegan diet for >1 year were recruited to donate a single stool sample. Participants ate their normal diets and donated a morning fecal sample in stool hats (Fisher Scientific, Hampton, NH, USA). The fecal samples were transferred to conical tubes and stored at −80 °C until further processing. The work involving human samples was carried out in accordance with the Sanford Burnham Prebys Medical Discovery Institute Institutional Review Board and guidelines for the study of human subjects. All the subjects gave written informed consent in accordance with the Declaration of Helsinki. The protocol was approved by the Sanford Burnham Prebys Medical Discovery Institute’s Institutional Review Board (IRB-2014-020).

### 2.2. Nervine Medicinal Herbs Examined in the Current Microbiome Study

We examined 10 medicinal herbs in this study which were all sourced from Banyan Botanicals (Albuquerque, NM, USA), with the exception of Jatamansi, which was sourced from AyurOrganics (Victoria, Australia).

### 2.3. Anaerobic Fecal Cultures

The stool samples derived from subjects with PD were used to inoculate chemically defined medium (CDM) or CDM that was supplemented with medicinal herbs (1% *w/v*). Approximately 10^6^ cells were introduced into CDM to allow multiple doublings before achieving saturation. The healthy stool samples used for comparison were derived from an equal volume mixture of stool collected from 12 healthy vegetarian participants as a fecal pool. Cultures were grown under anaerobic conditions (9% H_2_, 10% CO_2_, 81% N_2_) statically for 2 days at 37 °C. Healthy fecal pools were grown as technical replicates (n = 4–6), whereas PD stools were not. All the cultures were grown to approximate saturation and were then harvested by centrifugation. The recovered material was used immediately for genomic DNA isolation.

### 2.4. Chemically Defined Medium

CDM contains 50 mM N-2-hydroxyethylpiperazine-N′-2-ethanesulfonic acid (HEPES), 10 mM Na_2_HPO_4_, 60 mM NaHCO_3_, 4 mM of each amino acid except leucine (15 mM), 2.2 mM KH_2_PO_4_, and 10 mL ATCC Trace Mineral Supplement. CDM contained inosine, xanthine, adenine, guanine, cytosine, thymidine and uracil (400 mg/L) and nucleoside bases (100 mg/L). CDM contained ascorbic acid (500 mg/L), myo-inositol (400 mg/L), choline (100 mg/L), lipoic acid (2 mg/L) and hemin (1.2 mg/L). Resazurin (1 mg/L) was added to visually monitor the dissolved oxygen. The pH of the media was adjusted to 7.4. Medicinal herbs (2% *w/v*) in sterile water and 2× CDM were pre-reduced separately in an anaerobic chamber (Coy Labs) for 2 days. Equal volumes of medicinal herb and 2× CDM were combined just prior to inoculation to achieve a final medium: 1× CDM containing 1% medicinal herb.

### 2.5. Microbial DNA Isolation

Genomic DNA was isolated from the cultures and the fecal inoculum using the procedures of the QiaAmp DNA stool kit (Qiagen, Germantown, MD, USA) with a modification that included an additional step of bead beating using the Thermo FastPrep instrument (MP Bio, La Jolla, CA, USA) to ensure the uniform lysis of bacterial cells. The DNA was purified with QIAquick (Qiagen) purification kit columns. DNA integrity was analyzed using spectrophotometry and visualized using gel electrophoresis. Quantitative PCR was used to allow equivalent amounts of each amplicon generated in each sample to be pooled for library construction.

### 2.6. 16S rRNA Sequence Analysis

Multiplexed 16S rRNA libraries were prepared using standard 16S rRNA metagenomic sequencing library protocols from Illumina, which uses the V3-V4 region of 16S rRNA for target amplification and subsequent analysis. We used Qiime 2 for all the taxonomic analyses at all taxonomic levels. Raw sequence reads were filtered, denoised and paired read merged, and the chimeras were removed using default parameters in dada2 [37] to generate abundance tables with amplicon sequence variants (ASVs) representing unique 16S rRNA sequences. We used the multi-taxonomy approach (MTA) to assign taxonomic descriptions. Each ASV sequence was aligned with the 16S rRNA sequences present in the Ribosomal Database Project (RDP, version 11.5). The alignments obtained were sorted by the percent identity of the best matches, and the maximum values were denoted as M. We then collected and processed the taxonomic assignments for the identified 16S rRNA sequences with identities higher than the threshold M–(1–M)/4. The resulting multi-taxonomy assignments consisted of one or more taxonomic names separated by “/” where a higher resolution was not possible. To account for variable 16S rRNA gene copy numbers in reference genomes, we renormalized each sample’s ASV abundance by the average 16S rRNA copy number at each taxonomic level provided by the rrnDB database [38]. The obtained taxonomic profiles were further used for predictive genome-wide metabolic reconstruction for 16S rRNA metagenomic samples, as described below.

### 2.7. Genome-Wide Metabolic Reconstruction

We used a subsystem-based approach using microbial community SEED [39] to predict the metabolic potential of microbial taxa identified, as described previously [34]. The mcSEED metabolic subsystems curated for >2800 human gut microbial genome metabolic subsystems included biochemical pathways classified into two categories: (1) the biosynthesis of vitamins [40], amino acids and cofactors [41] and (2) the production of SCFAs [42]. The pathways of interest were categorized using binary scoring, where they were assigned a “1” or a “0” for the presence or absence of a pathway, respectively. The binary phenotype matrix (BPM) obtained for the metabolic phenotype distributions in the reference genomes was used to calculate a community phenotype matrix for all the enumerated taxa obtained from the 16S rRNA analysis. The community phenotype index (CPI) for each metagenomic sample and metabolic phenotype was calculated using a development version of the Phenotype Profiler tool provided by PhenoBiome Inc. (Palm Springs, CA, USA), as previously described [43].

## 3. Results

We anaerobically cultivated each of the three PD subject’s stool samples in CDM or CDM supplemented with one of 10 nervine medicinal herbs (Table 1).

### 3.1. Diversity and Modulatory Effects of Medicinal Herbs

**Figure 1:**
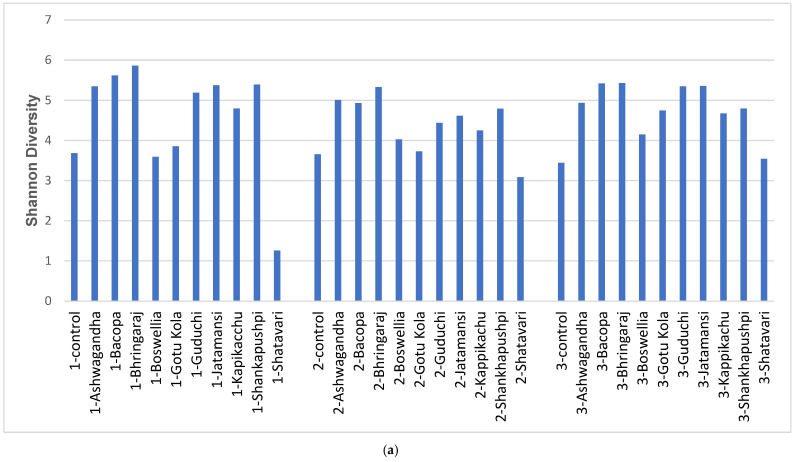

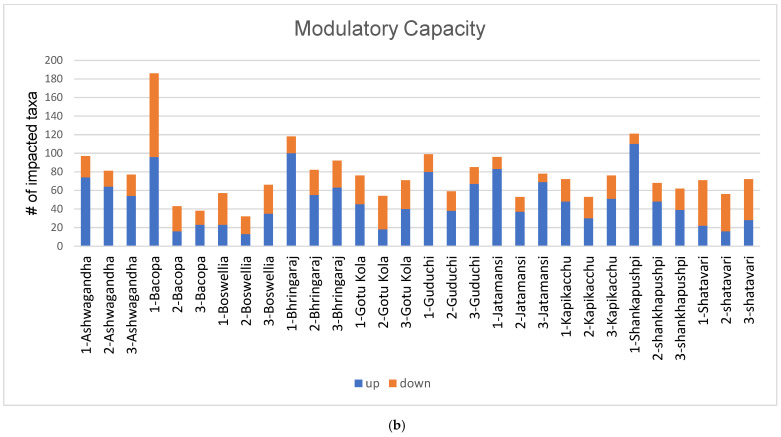
Microbiota diversity and the modulatory potential of medicinal herbs. (**a**) Shannon α diversity measurements of PD stool with and without medicinal herb supplementation. (**b**) Species-level taxa altered by nootropic herbs using a 5-fold change as a cut-off for increased/decreased relative abundance.

It has been reported that PD gut microbiota displays reduced α diversity [2,4,11,44,45,46]. Therefore, we analyzed Shannon diversity in control and herb-supplemented cultures derived from PD stool (Figure 1a).

Several nervine herbs increased the α diversity of communities in all three subjects, including Ashwagandha, Bhringaraj, Guduchi, Jatamansi, Kapikacchu and Shankhapushpi, whereas in subject 3, α diversity was also increased by Boswellia and Gotu Kola. Shatavari resulted in a reduced α diversity in all three subjects. Stool derived from healthy subjects was not included in this analysis because this sample is a fecal pool generated from 12 subjects with inherently higher α diversity. We assessed the modulatory capacity of each nervine herb to compare herb responsiveness across the subjects (Figure 1b; Supplementary Table S2).

Overall, subject 1 displayed the greatest response to nervine herb supplementation, with the average modulatory capacity across all 10 medicinal herbs showing an increase in relative abundance for 68 taxa and a decrease for 31 taxa, compared to 34 taxa showing an increase and 25 showing a decrease in subject 2, and 47 showing an increase and 25 showing a decrease in subject 3. Bhringaraj had the greatest modulatory capacity across all PD subjects and Boswellia had the least impact on resident taxa. Bhringaraj and Shankhapushpi displayed the greatest inter-personal variability in their responsiveness to medicinal herbs, followed by Bhringaraj, Guduchi and Jatamansi.

### 3.2. Phylogenetic Analysis and Inter-Personal Variable Responses to Nootropic Herbs

At the phylum level, nearly all medicinal herb supplementation resulted in the reduction of Firmicutes, Proteobacteria and Actinobacteria (Supplementary Table S3). Exceptions in these trends included Guduchi, Jatamansi and Shatavari in subject 3, where Firmicutes were increased. Proteobacteria were increased in all the samples supplemented with Gotu Kola, subjects 1 and 2 supplemented with Boswellia and subject 2 supplemented with Jatamansi. Gotu Kola (subject 3) and Guduchi (subject 1) supplementation led to an increased representation of Actinobacteria. All the herb treatments resulted in an increased representation of Bacteroidetes in all the subjects.

At the family level, we observed a greater divergence in herb responsiveness across the subjects (Supplementary Table S4). Bacteroidaceae and Tannerellaceae were uniformly increased in all the subjects by all the treatments, whereas an unclassified Flavonifactor family was reduced in relative abundance in all the subjects by all the treatments. With one exception (Gotu Kola, subject 3), Eggerthellaceae were also uniformly decreased in all the subjects by all the treatments. Among the remaining families of high to moderate abundance, medicinal herb supplementation resulted in mixed results in terms of the relative abundance across herbs, subjects or both.

### 3.3. Nootropic Herb Supplementation Corrects Genus-Level Dysbioses Associated with PD

A meta-analysis of 13 microbiota profiling studies derived from PD subjects from Italy, USA, Finland, Canada, Russia, Malaysia, Germany, China, Japan, Luxembourg and Australia provided a consensus of impacted taxa, avoiding potential country-specific effects [47]. We examined these differentially related taxa in nervine herb supplemented cultures to determine their potential to correct the dysbioses apparent in PD subjects (Table 2; Supplementary Table S5).

In total, 27 genera displayed differential relative abundance that was either increased or decreased in a majority of studies. Among the ten medicinal herbs tested, PD-associated dysbioses were not corrected in healthy subjects or any of the three PD subjects for five genera, including *Butyrivibrio*, *Catabacter*, *Desulfovibrio*, *Enterobacter* and *Parabacteroides.* This result was not due to the inability to cultivate these taxa. Beyond these cases, no taxa responded uniformly to herb treatments for these potentially important genera. An assessment of the individualized responses to medicinal herbs showed that healthy subjects responded in a manner (direction of change) to correct PD dysbiotic genera in 31% of herb treatments, which was higher than that of individual PD subjects. In subject 1, 26% of the treatments resulted in corrective increased/decreased relative abundance of the genera by at least 3-fold. These values were lower for subjects 2 and 3, respectively (20% and 22%). It is notable that a number of taxa underrepresented in PD fecal microbiota were undetected in any culture condition, including *Gemmiger* (subjects 1 and 3), *Coprococcus* (subjects 2 and 3), *Fusicatenbacter* and *Lachnospira* (subject 3). It is therefore possible that these taxa could not be “rescued” by medicinal herbs due to their absence in these subjects. In support of this interpretation, no taxa of interest were absent in healthy subject pools.

Overall, Ashwagandha and Kapikacchu displayed the greatest corrective potential across all the subjects, involving a 32% and 31% correction of relevant genera, respectively. For subject 1 and 2, Ashwagandha displayed correction potential for 37% and 30% of the relevant genera, respectively. The best outcomes for subject 3 were observed with Ashwagandha and Bacopa, both impacting 30% of the relevant genera. Given the relative importance of *E. coli* produced LPS as a promoter of systemic inflammation and microglial activation in PD, we noted that Ashwagandha, Bhringaraj, Kapikacchu and Shatavari treatments drove reductions in the relative abundance of *E. coli* in all the subjects.

### 3.4. Interpersonal Variability of PD Microbiota to Medicinal Herbs

Among the 265 taxa profiled across the three PD subjects, only a small fraction responded uniformly across all the three subjects to a single medicinal herb treatment (Supplementary Table S6). The cultures supplemented with Bhringaraj displayed the greatest uniformity with 29 taxa (11% of total), whereas Gotu Kola had the least uniformity, involving only 3% of profiled taxa. These results highlight the substantial personalized response to medicinal herbs as it pertains to gut microbiota modulation. The number of taxa displaying uniform directions of change increased as expected when the criteria were relaxed to include two out of the three subjects. Bacopa supplementation generated the highest uniformity, involving 26% of the profiled taxa. It is notable that the majority of taxa displaying uniform change by either criterion displayed increased relative abundance; however, Shatavari supplementation generated the greatest number of taxa displaying reduced relative abundance, involving 4% of taxa shared between all three subjects and 11.7% of taxa shared between at least two subjects.

### 3.5. Gut Permeability and PD Microbiome

**Figure 2:**
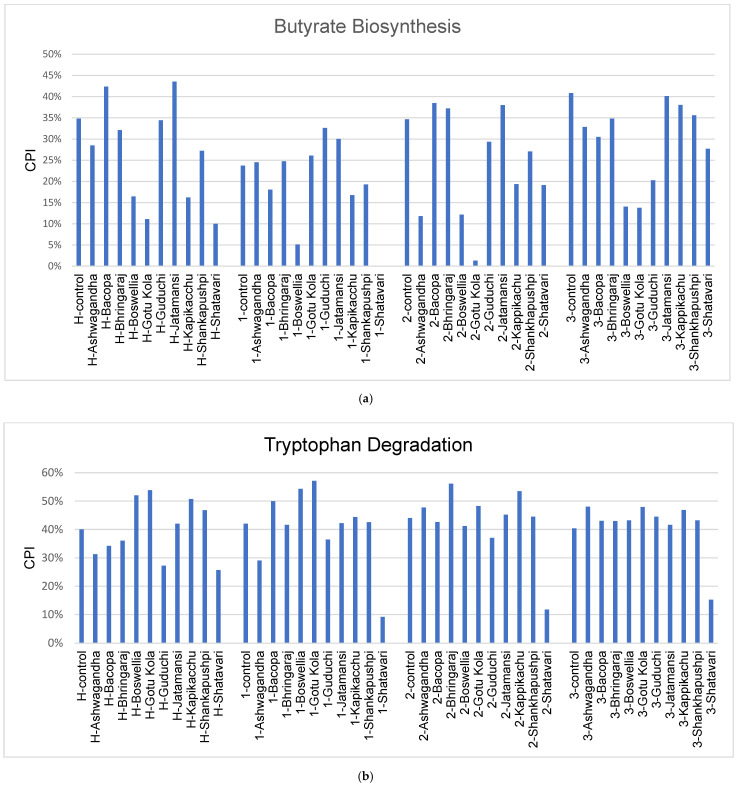
Microbial metabolites that decrease gut permeability in medicinal-herb-treated cultures. (**a**) Community Phenotype Index (CPI) for butyrate biosynthesis. (**b**) CPI for tryptophan degradation.

Several reports have indicated that subjects with PD display increased gut permeability [12,48,49]. This phenotype is likely to be of high significance in disease progression as elevated gut permeability is associated with a higher translocation of LPS across the gut epithelium, which leads to increased activation of inflammatory cytokines and microglial activation in the CNS. Currently, there is no FDA approved drug to reduce gut permeability; however, a growing consensus of in vitro and rodent models indicates that butyrate acting via the GPR109A receptor and tryptophan degradation products (indoles) acting through the arylhydrocarbon receptor decrease gut permeability [50,51,52]. We used genome-wide metabolic reconstruction to calculate the relative abundance of microbial community members capable of producing butyrate and tryptophan degradation products (Figure 2a). For each metabolic pathway and sample, we calculated a Community Phenotype Index (CPI) for comparison across individuals.

In healthy subjects, Bacopa and Jatamansi treatments led to an increased CPI for butyrate biosynthetic potential, whereas Guduchi and Jatamansi increased these pathways in subject 1. In subject 2, Bacopa, Bhringaraj and Jatamansi treatments increased the CPI for butyrate. No medicinal herbs resulted in increased butyrate potential in subject 3. This result is most likely due to the high predicted butyrate biosynthetic potential displayed in control cultures.

The analysis of tryptophan degradation pathways showed that Boswellia increased the CPI for tryptophan degradation in healthy subjects and in subject 1, whereas Gotu Kola did the same in healthy subjects and in subjects 1 and 3. Kapikacchu treatment increased the CPI for tryptophan degradation in healthy subjects and in subjects 2 and 3. Shankhapushpi uniquely increased the CPI in healthy subjects, whereas Bacopa (subject 1), Bhringaraj (subject 2) and Ashwagandha (subject 3) uniquely elevated community representations of this pathway (Figure 2b).

### 3.6. Vitamin Biosynthesis Potential

**Figure 3:**
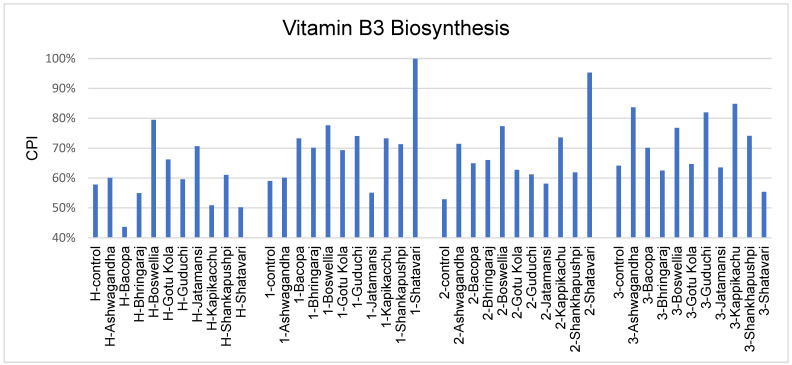
Community-wide metabolic potential for vitamin B3 biosynthesis in medicinal-herb-treated cultures.

Several vitamin deficiencies have been linked to patients with PD. The greatest focus has been on vitamin B3 (niacin) due to its neuroprotective effects [53]. At least three medicinal herb treatments were able to increase the vitamin B3 biosynthetic potential of gut microbiota in healthy and PD subjects, although the specific medicinal herb inducing this effect differed across the subject groups (Figure 3).

**Figure 4:**
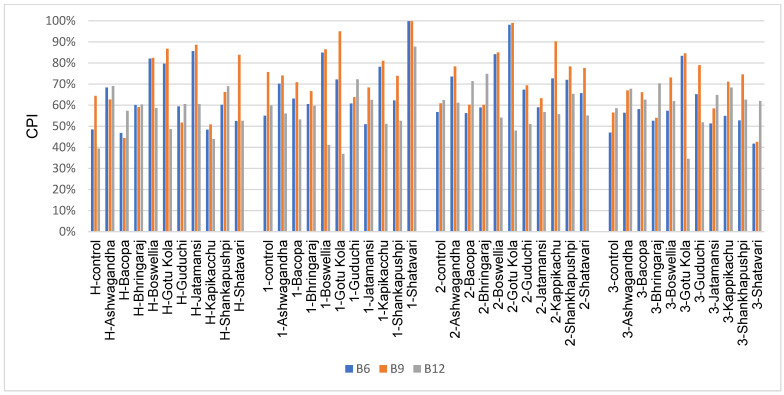
Community-wide metabolic potentials for the biosynthesis of other B vitamins in medicinal-herb-treated cultures.

It is estimated that 30% of PD patients exhibit elevated homocysteine levels, which is positively correlated with cognitive decline [54,55]. Homocysteine levels are linked to the B vitamins (B6, B9 and B12) of the homocysteine pathway. We examined the impact of medicinal herbs on the production of these B vitamins (Figure 4), which are known to be secreted and are therefore shared with the host [41,56].

Herb-induced increases in the biosynthetic potential of vitamin B6, B9 and B12 were noted for healthy and PD subjects in each case and displayed high subject-to-subject variability. In subject 1, Shatavari supplementation increased all three B vitamins substantially. In all the PD subjects, Gotu Kola increased the biosynthetic potential of B6 and B9, but not B12. Both Bacopa and Bhringaraj increased vitamin B12 in subject 2, whereas several medicinal herbs increased the biosynthetic potential of B12 in subject 3; however, these changes were more modest.

## 4. Discussion

The Braak hypothesis proposes that sporadic PD initiates in the gastrointestinal tract and within the neurons of the olfactory system [57]. A modification of this hypothesis suggests that PD begins in either the enteric nervous system or the olfactory system but rarely both [58]. The Braak hypothesis suggests the possible role of gut microbiota in the initiation and progression of PD. Conceptually, the myriad of metabolites produced by gut microbiota may impact PD via the vagus nerve or passage across the single cell epithelial layer of the gut. Indeed, gut permeability is elevated in PD patients, as is dysbiosis of the gut microbiota, which involves a relatively high fraction of species [13]. The combination of increased gut permeability and *E. coli* is of particular importance.

Drug development efforts are actively being pursued to treat aspects of PD. Natural therapies that normalize disease phenotypes are also in demand. In this study, we demonstrate that the impact of nootropic herbs on fecal microbiota is personalized. Despite the high subject-to-subject variability in response to medicinal herbs, we provide a proof of concept through the use of in vitro cultivation of patient-derived fecal samples with a panel of 10 nootropic herbs to enable rational choices that are intended to provide personalized treatment to patients to best target disease phenotypes.

### 4.1. Diversity and Modulatory Effects of Medicinal Herbs

The impact of medicinal herbs on fecal microbiota was apparent in all the subjects. Several herbs increased the α diversity of communities (Figure 1a). Most of the herbs tested increased α diversity in all three subjects; however, the magnitude of change was personalized. Similarly, all the medicinal herbs displayed substantial modulatory capacity, which primarily resulted in increased relative abundance of the impacted taxa, although Shatavari and Gotu Kola supplementation resulted in a greater number of taxa with reduced relative abundance in all three subjects (Figure 1b; Supplementary Tables S1 and S2). Subject 1 was more responsive to medicinal herbs and showed the greatest modulation of communities compared to the other subjects.

### 4.2. Nootropic Herb Supplementation Corrects Genus-Level Dysbioses Associated with PD

The analysis of taxonomic changes induced by medicinal herbs in PD subjects focused on the genera identified by a meta-analysis to be dysbiotic (Table 2; Supplementary Table S5). The greatest number of taxa altered in the proper direction to correct dysbioses was observed for healthy human donor stool (31%) compared to 20–26% in PD subjects (Supplementary Table S6). It is notable that a small number of the 27 genera observed as dysbiotic in the PD meta-analysis were apparently absent in the PD subjects tested in this study. These taxa were never observed in control cultures or any of the 10 medicinal herb supplemented cultures. It is unclear whether these subjects harbored uncultivable strains or if these genera were truly lost in the PD subjects as the result of the disease. 

Ashwagandha and Kapikacchu displayed the greatest “corrective” potential among the medicinal herbs tested. The personalized response to medicinal herb supplementation was best illustrated by the fact that at most, only 11% of the 265 taxa detected in all the PD subjects responded uniformly in response to Bhringaraj supplementation. The specific mechanism dictating personalized responses to medicinal herbs remains unclear; however, we speculate that strain-level variability in subjects may account for the disparate responses observed. It is also possible that higher-order community interactions involving complex cross-feeding behaviors contribute to personalized responses.

### 4.3. Gut Permeability and PD Microbiome

The human gut microbiome displays large fractions of Gram-negative bacterial species that encode lipopolysaccharide (LPS) in their cell wall. The vast majority of Gram-negative bacteria encode a penta-acylated LPS, which signals through TLR4 receptors to stimulate healthy inflammatory tone [59]. By contrast, hexa-acylated LPS, which is encoded by *E. coli* and its close relatives, is highly pro-inflammatory, stimulating cytokine production which reinforces gut permeability and systemic inflammation. In the context of PD, circulating LPS is thought to play a key role in the activation of microglial cells in the CNS that accompany disease progression [60].

Among the known metabolites produced by gut microbes, butyrate and a variety of tryptophan degradation products (indoles) are known to decrease gut permeability. The nootropic herb effects on butyrate and tryptophan degradation were varied across the subjects (Figure 2a). Jatamansi supplementation increased butyrate in healthy subjects and in PD subjects 1 and 2, but due to predicted high control butyrate levels, no medicinal herbs further increased the biosynthetic potential of butyrate in subject 3. We also noted several medicinal herbs that increased tryptophan degradation potential (Figure 2b). Gotu Kola and Bhringaraj increased the potential of both butyrate and tryptophan degradation products in subjects 1 and 2, and Kapikacchu increased the potential of butyrate and tryptophan degradation products in subject 3.

### 4.4. Vitamin Biosynthesis Potential

The levels of niacin in PD patients are reduced due to the use of dopaminergic drugs that prevent the conversion of tryptophan to niacin [61]. In addition to being a neuroprotective agent, niacin also acts to reduce LPS-induced microglial activation [62]. Medicinal herb supplementation increased the biosynthetic potential of niacin in a high fraction of medicinal herbs tested; however, the specific herbs differed across the subjects (Figure 3).

Methionine metabolism (demethylation) leads to homocysteine production. Homocysteine elimination (level reduction) is achieved through various chemical modifications mediated by enzymes that use vitamin B6, B9 or B12 as co-factors [55]. Therefore, deficiencies in these B vitamins can exacerbate homocysteine-induced cognitive decline. Several medicinal herbs increased the biosynthetic potential of vitamin B6 and B9; however, increases in vitamin B12 were rarer. Consequently, only Shatavari supplementation in subject 1 increased the biosynthetic potential of all three B vitamins.

### 4.5. Limitations and Conclusions

This work illustrates the potential utility of nootropic herbs used in Ayurvedic medicine to modulate the structure and function of the gut microbiota. Compared to pure prebiotic fibers, such as inulin, GOS and others, we recently showed that medicinal herbs have a greater impact on gut microbiota due to the complexity of their constituent glycan content [63]. In addition to their effects on gut microbiota, nootropic herbs have additional qualities that may alleviate PD symptoms, such as anxiety and constipation. Based on previous studies, we and others have noted that microbiota modulatory substances lead to highly personalized alterations that increase the challenge of using these treatments as part of patient therapy.

This study included a small number of participants, limited in part by the COVID-19 pandemic and the need to limit the number of samples to be screened under 11 different culture conditions. Although our conclusions based on this proof-of-concept experimentation are limited to three PD patients, the magnitude of the differences across individuals suggests that it is unlikely that our conclusions are invalid. This work re-confirms the personalized nature of gut microbiota responses to medicinal herbs, but also illustrates the potential utility of using patient stool as a means of prioritizing the medicinal herbs with the greatest likelihood of targeting the phenotypes of interest. Although the specific relationship between the in vitro cultivation of herb-supplemented stool samples and their effects in humans has not been determined, these in vitro results help to define the capacity of patient-specific strains to respond to medicinal herb constituents. Additional human intervention trials coupled with in vitro cultivation will be required to fully test this proof of concept for personalized therapies and clinical strategies featuring medicinal herbs and prebiotics to achieve desired clinical outcomes.

## Figures and Tables

**Table 1:** Nootropic Herbs studied. Selected common names and family information are shown. Note that in some regions in India, brahmi may refer to *C*. *asiatica*. In addition, shankhapushpi may refer to any of four nervine species, including *Convulvulus pluricaulis* (Convulvulaceae), *Evolvulus alsinoides* (Convulvulaceae), *Clitoria ternatea* (Papilionaceae) and *Canscora decussata* (Gentianaceae); the commonly used *E*. *alsinoides* was examined here.

Species	Common Name	Family
*Asparagus racemosus*	Shatavari	Asparagaceae
*Bacopa monnieri*	brahmi or waterhyssop	Plantaginaceae
*Boswellia serrata*	Frankincense	Burseraceae
*Centella asiatica*	gotu kola or pennywort	Apiaceae
*Eclipta alba*	bhringaraj or false daisy	Asteraceae
*Evolvulus alsinoides*	shankhapushpi	Convolvulaceae
*Mucuna pruriens*	kapikacchu or velvet bean	Fabaceae
*Nardostachys jatamansi*	Jatamansi	Valerianaceae
*Tinospora cordifolia*	Guduchi	Menispermaceae
*Withania somnifera*	Ashwagandha	Solanaceae

These cultures were used to purify genomic DNA that then served as a template for amplification of the V3-V4 region of the 16S rDNA locus. The 16S sequence data was used to enumerate the taxa in the cultures for comparative analysis (Supplementary Table S1). In total, this analysis resulted in the enumeration of 265 taxa, with most of them resolved to the species level.

**Table 2:** Corrective potential of medicinal herbs for PD dysbiosis.

Taxa	PD>HC	PD<HC	Ashwagandha	Bacopa	Boswellia	Bhringaraj	Gotu kola	Guduchi	Jatamansi	Kapikacchu	Shankhapushpi	Shatavari	1	2	3	H	Average
Akkermansia	13	0	2,3,H	2,3,H	H	2,3,H	H	2,3,H	2,H	H	2,3,H	2,3,H	0%	70%	60%	100%	58%
Alistipes	5	0	3	2	1,2.3,H	none	1,2	2	2	2,3,H	2	1,2,3	30%	80%	40%	20%	43%
Anaerotruncus	2	0	1,H	none	none	none	1,H	H	none	1,H	H	1,H	40%	0%	0%	60%	25%
Anaerotruncus/C. Soleaferrea/other	2	0	none	none	none	none	H	none	none	H	H	H	0%	0%	0%	40%	10%
Bacteroides	1	2	1,2,3	1,2,3	1,2,3,H	1,2,3	1,2,3,H	1,2,3	1,3	1,2,3	1,2,3,H	1,H	100%	80%	80%	40%	75%
Bifidobacterium	10	0	2	2	2	none	2,H	none	none	none	none	2	0%	50%	0%	0%	13%
Bilophila	4	0	none	none	none	none	none	2	none	none	none	2	0%	20%	0%	0%	5%
Blautia	0	7	2	none	none	none	none	2	2	none	none	none	0%	30%	0%	0%	8%
Butyrivibrio/Fusicatenibacter/Pseudobutyrivibrio	2	1	none	none	none	none	none	none	none	none	none	none	0%	0%	0%	0%	0%
Catabacter	2	0	none	none	none	none	none	none	none	none	none	H	0%	0%	0%	0%	0%
Christensenella	3	0	none	none	H	H	none	none	none	H	none	H	0%	0%	0%	40%	10%
Coprococcus	1	2	1,2,3,H	3,H	2,3	1,3,H	1	1,3,H	1,3	2,3,H	1,3	none	60%	30%	80%	50%	55%
Coprococcus/Frisingicoccus	1	2	1	1	none	none	none	none	none	none	none	none	20%	0%	0%	0%	5%
Desulfovibrio	3	0	none	none	none	none	none	none	none	none	none	none	0%	0%	0%	0%	0%
Dorea	0	2	2,3	2,3	none	2,3	3	2,3	2,3	2,3	none	none	0%	60%	70%	0%	33%
Enterobacter	2	0	none	none	none	none	none	none	none	none	none	none	0%	0%	0%	0%	0%
Enterococcus	3	0	none	none	none	none	3	3	3	none	3	none	0%	0%	40%	0%	10%
Escherichia	3	0	1,2,3,H	1,3,H	H	1,2,3,H	H	1,H	1,H	1,2,3,H	1,H	1,2,3,H	80%	40%	50%	100%	68%
Faecalibacterium	0	8	1,H	1,3,H	1,H	1,H	1,H	1,H	1,H	1,H	1,H	1	100%	0%	10%	90%	50%
Fusicatenibacter	0	3	1,H	H	none	H	H	H	none	1,2,H	none	none	20%	10%	0%	60%	23%
Gemmiger	0	2	2,H	H	2,H	2,H	H	H	2,H	2,H	2,H	none	0%	60%	0%	90%	38%
Holdemania	2	0	1,3	none	1,3	none	1	none	none	3	none	1,3	40%	0%	40%	0%	20%
Lachnospira	1	2	1,H	1,H	none	1,H	none	1,H	1,H	1,H	1,H	1	80%	0%	0%	70%	38%
Lactobacillus	9	2	3	3	3	3	3	3	3	3	3	3	0%	0%	100%	0%	25%
Parabacteroides	3	1	none	none	none	none	none	none	none	none	none	none	0%	0%	0%	0%	0%
Roseburia	1	9	1,H	1,3,H	none	1,H	none	1,H	1,3,H	1,3,H	1,H	none	70%	0%	30%	70%	43%
Ruminococcus	1	2	none	1	none	1	none	1	none	1	1	none	50%	0%	0%	0%	13%
												Average	26%	20%	22%	31%	25%
												Total	690%	530%	600%	830%	663%

## Data Availability

The 16S rRNA sequence data for PD subjects is available under BioProject: PRJNA988266. The healthy stool 16S rRNA sequence data published previously may be found at NCBI under BioProject: PRJNA497131.

## Supplementary Materials

The following supporting information can be downloaded at: https://www.mdpi.com/article/10.3390/microorganisms11081979/s1, Table S1: 16S rRNA sequence enumeration; Table S2: Fold change of taxa by medicinal herbs; Table S3: Phylum level changes in herb-supplemented cultures; Table S4: Family level changes in herb-supplemented cultures; Table S5: Dysbiotic taxa in PD response to medicinal herbs; Table S6: Uniformity of subjects to nootropic herbs.
